# Development and quality assessment of the psychometric properties of the Self-Efficacy in Lifestyle Counselling scale (SELC 20 + 20) using Rasch analysis

**DOI:** 10.1186/s12955-024-02236-z

**Published:** 2024-02-23

**Authors:** Sara Alenius, Albert Westergren, Petra Nilsson Lindström, Marie Nilsson, Marie Rask, Lina Behm

**Affiliations:** 1 Faculty of Health Sciences, Department of Nursing and Integrated Health Sciences, Kristianstad University, Kristianstad, Sweden; 2 The PRO-CARE Group and The Research Platform for Collaboration for Health, Faculty of Health Sciences, Kristianstad University, Kristianstad, Sweden

**Keywords:** Counseling, Health Promotion, Life Style, Psychometrics, Quality of Health Care, Self-efficacy, Students Nursing, Surveys and Questionnaires, Sweden

## Abstract

**Background:**

Globally as well as in Sweden, diseases that are caused by unhealthy lifestyle habits are the most common causes of death and disability. Even though there are guidelines that oblige all health-care professionals to counsel patients about lifestyle, studies have shown that it is not prioritized within healthcare. One reason for this among nurses has been shown to be lack of confidence in knowledge and counselling skills. This study aimed to develop, and quality assess the psychometric properties of an instrument to measure self-efficacy in lifestyle counselling.

**Methods:**

An instrument inspired by an American instrument, following Bandura’s recommendations for development of self-efficacy measures, was developed according to Swedish national guidelines for disease-prevention. The instrument was revised after cognitive interviews with nursing students, university teachers within health sciences, and clinical experts, then administrated to 310 nursing students at different levels in their education. The instrument was tested with Rasch Measurement Theory, with focus on dimensionality, local dependency, targeting, reliability, response category functioning, Rasch model fit, and differential item functioning by age, gender, educational level and previous health care education.

**Results:**

The development of the instrument resulted in 20 + 20 items, 20 items about self-efficacy in knowledge, and 20 items about self-efficacy in ability to counsel persons about their lifestyle. The analyses showed that knowledge and ability are two different, but related, constructs, where ability is more demanding than knowledge. The findings provide support (considering dimensionality and local dependency) for that all 20 items within the knowledge construct as well as the 20 items within the ability construct can be summed, achieving two separate but related total scores, where knowledge (reliability 0.81) is a prerequisite for ability (reliability 0.84). Items represented lower self-efficacy than reported by the respondents. Response categories functioned as expected, Rasch model fit was acceptable, and there was no differential item functioning.

**Conclusions:**

The SELC 20 + 20 was found to be easy to understand with an acceptable respondent burden and the instrument showed good measurement properties.

**Supplementary Information:**

The online version contains supplementary material available at 10.1186/s12955-024-02236-z.

## Background

Health promotion and disease prevention are cost-effective ways of reducing premature death and disability, as well as increasing the quality of life [1, 2]. Globally as well as in Sweden, non-communicable diseases (NCDs) are one of the most common causes of death and disability [3]. The most common NCDs, such as cardiovascular disease, some types of cancer, diabetes type 2, and chronic lung disease are often caused by unhealthy lifestyle habits [4–7]. The four habits that influence the risk of NCDs the most are tobacco use, alcohol consumption, insufficient physical activity and unhealthy eating habits [8]. In Sweden, it has since the 1980s been regulated by law that health care professionals (e.g. nurses, assistant nurses, medical doctors, paramedics and public health practitioners) shall give disease preventing advice to all patients who can benefit from it [9]. Population surveys show that most patients are positive about lifestyle counselling in health care [10, 11]. However, only 32% of all patients in Sweden receive lifestyle counselling [12], which appears to be due to barriers among healthcare professionals to carrying out lifestyle counselling. Since 2018 the National Board of Health and Welfare demands all healthcare professionals to counsel patients about the lifestyle habits: tobacco use, alcohol consumption, physical activity and eating habits in all encounters within healthcare [13].

Regarding doctors and nurses, the literature presents barriers for carrying out lifestyle counselling such as a lack of counselling skills, lack of confidence, concerns about the effectiveness of their counselling as well as lack of time [14–22]. Many of the barriers for healthcare professionals to carry out lifestyle counselling described in literature originate in low counselling self-efficacy [14–22]. Bandura defines self-efficacy as “the confidence to carry out the courses of action necessary to accomplish desired goals” [23]. Self-efficacy is not a general trait, it is context-specific [24]. It is not necessarily dependent on a person’s skills, but rather on the person’s confidence in their ability to use the skills they have in a given situation. Low self-efficacy can hinder despite a high level of knowledge, as well as a high self-efficacy cannot compensate for a lack of knowledge [23]. Four information sources affect self-efficacy: *actual performances* (e.g. challenging tasks with support), *vicarious experiences* (e.g., seeing others succeed at the task), *forms of social persuasion* (e.g., feedback and encouragement) and, *physiological information* (e.g. minimizing anxiety while performing the task) [23, 25]. Self-efficacy consists of three dimensions: *magnitude* (e.g., perceived level of difficulty), *strength* (e.g., how confident the respondent is) and *generality* (e.g., if and how the self-efficacy beliefs are positively related across domains or time) [26]. Instruments to measure self-efficacy need to be task-specific and optimally include all three dimensions [23, 27]. For lifestyle counselling self-efficacy, theoretical knowledge about lifestyle habits as well as practical ability to counsel patients are needed [28].

Although many of the barriers for healthcare professionals to carry out lifestyle counselling originate in low self-efficacy, and self-efficacy has been shown in research [29] to be an outcome to be used in lifestyle counselling research and practice there is, to our knowledge, no instrument to measure self-efficacy in lifestyle counselling that matches the Swedish national guidelines for disease-prevention: tobacco use, alcohol consumption, physical activity and eating habits. A literature search resulted in the identification of one American instrument called the Health Promotion Counselling Self-Efficacy Scale (HPCSES), developed by Tresolini et al. [28]. The HPCSES measures health promotion counselling self-efficacy in the five health domains: smoking, exercise, nutrition, sexually transmitted diseases and injuries. The instrument was considered as a relevant base for the development of a new instrument which focus on the lifestyle habits addressed in the national guidelines. An instrument that matches the Swedish national guidelines for disease prevention could be a helpful tool in the educations of healthcare professionals, as well as clinically in all areas of healthcare. Both to assess if there is a need for training in lifestyle counselling and to evaluate if a course or training increase self-efficacy. Initially, this instrument will be used to evaluate nursing students lifestyle counselling self-efficacy before and after clinical training.

## Methods

### Aim

This study aimed to develop, and quality assess the psychometric properties of an instrument to measure self-efficacy in lifestyle counselling.

### Design

This study was divided into two parts, the first part was the development of the instrument, and the second part was a cross-sectional study with purposive sampling to psychometrically evaluate the newly developed instrument.

## Part 1: Development of the instrument

**Fig. 1:**
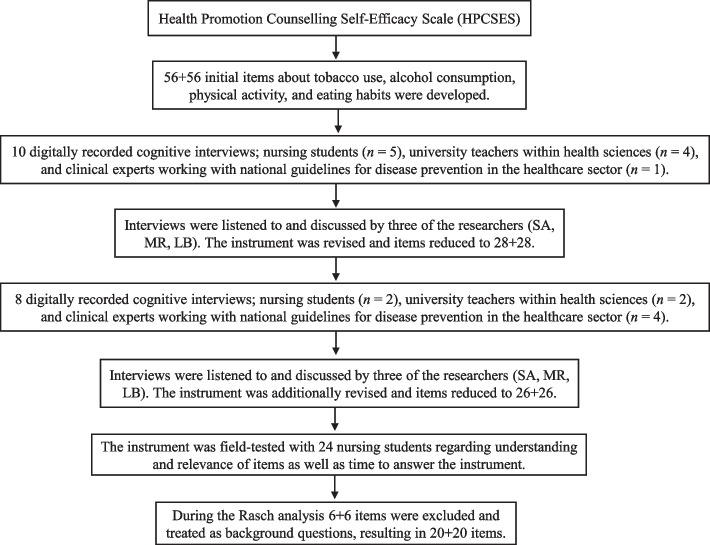
Flowchart illustrating the development of the instrument

The existing scientific literature showed a need for an instrument to measure self-efficacy in lifestyle counselling about tobacco use, alcohol consumption, physical activity and eating habits. In December 2019 on of the authors (LB) received a permission to develop an instrument inspired by the HPCSES from Tresolini (Fig. 1).

Initially, 56 + 56 items were developed by the research group in accordance with the national guidelines for disease-prevention methods in Sweden [13]. The instrument includes items about theoretical knowledge about-, and practical ability to counsel patients in the four lifestyle domains: tobacco use, alcohol consumption, physical activity, and eating habits, and measures lifestyle counselling self-efficacy. A 4-point semantically anchored Likert-scale was used (1 = I am very insecure in my…, 2 = I am insecure in my…, 3 = I am sure of my…, 4 = I am very sure of my…). Cognitive interviews with purposefully sampled informants were conducted to make the instrument as clear and understandable as possible (e.g. How relevant do you think this question is? Was anything in the question hard to understand?), following the recommendations by Willis and Wenemark [30, 31]. The instrument was field-tested with 24 nursing students regarding how easy the instructions and items were to understand, the relevance of the items and how long time it took to answer the instrument. Lastly, 6 + 6 items relating to learning methods were also field-tested by the nursing students. These items are not included in the final instrument measuring self-efficacy in lifestyle counselling but could function as add-on items in intervention studies. Results in relation to these items will be presented in a future article.

## Part 2: Psychometric testing of the instrument

### Sample and data collection

In the psychometric testing of the instrument, nursing students at a university in southern Sweden, were invited to participate. In January 2023 all nursing students in semesters 2 to 6, physically present at the university, were asked to participate after an oral presentation about the study by a PhD student (SA). The instrument was handed out to everyone and those who did not want to participate handed in their blank instrument. In total 310 (89%) out of 347 students chose to participate. The mean (SD) age was 28 (7.5) and 87.7% (*n* = 272) were women, 12.9% (*n* = 40) had a previous university degree and 37.4% (*n* = 116) had previous health care education, for example assistant nurse.

### Rasch measurement theory

The early work within the field of psychometrics, the science of rating scales, is nowadays termed Classical Test Theory (CTT). Methods within CTT are typically based on correlations and on between person differences to define the attribute structure [32], not the measurement mechanism at the individual level. Findings from CTT studies are distribution-dependent and cannot be generalised beyond the characteristics of the sample used in the study. In CTT the raw summed total score, although it is ordinal, is regarded as a “measure” of the latent variable. Within the Modern Test Theory (MTT) paradigm there are methods that provides a deeper understanding and uncovers what is otherwise “hidden” by the correlation based CTT. Within MTT, as well as within clinimetrics [33–35], we have the Rasch measurement theory (RMT) that was developed by George Rasch (1960). RMT is a mathematical model to test the observed data to the measurement model [36]. The model estimates item location and person location separately on a common interval level logit (log-odd units) scale, ranging from minus to plus infinity with the mean item location at zero [37]. If data fit the model, then linear measurement and invariant comparisons are possible [38]. Thus, in opposite to CTT, one can through RMT ensure that between person differences have the same structure as within person differences. A sample around *n* = 250 to 500 is optimal for psychometrical analysis according to the RMT [39].

### Analysis

The analyses address dimensionality and local dependency, targeting, reliability, response category functioning, Rasch model fit, and Differential Item Functioning (DIF) by age (subgroups according to median age) gender, educational level and previous health care education [37, 40]. The data were analysed according to the unrestricted polytomous Rasch model using RUMM2030 (Version 5.8.1) [41, 42]. *P*-values (two-tailed) were considered significant when < 0.05 following Bonferroni adjustment [43].

#### Dimensionality and local dependency

One fundamental assumption of the Rasch measurement model is unidimensionality, that only one construct is being measured, in this case self-efficacy. Unidimensionality was assessed by means of principal component analysis of the residual correlations. Person location estimates were derived from two subsets of items, one that loaded positively and one that loaded negatively on the first principal component of residuals. Unidimensionality is supported if the overall proportion of persons with significantly different measures from the two item subsets is < 5% [44, 45].

Local dependency can occur because of response dependency and trait dependency. If different items relate to the same aspect of the construct (self-efficacy) there is a risk of response dependency, i.e. the response of one item can predict the response of another item. Individual residual correlations should preferably be compared to the average observed residual correlation instead of a uniform value [46]. The critical value for relative residual correlations in this study was identified as described by Christensen et al. [47]. Correlations higher than the critical value indicates local dependency. When the total score of an instrument consists of subscales, some local dependency within the same subscale is expected [37]. This can be accounted for by dividing the instrument into subtests according to the subscales, the items within the subscales are then treated as one single item in the analysis [37]. Local dependency can lead to an inflated estimate of reliability and if the reliability drops considerably in a subtest compared to the overall instrument, it is an indication of local dependency [46]. A low variance unique to the subscale (C^2^) combined with a high latent correlation (r) between the subscales and a high non-error variance (A) supports unidimensionality [48].

#### Targeting

Targeting helps to determine to what extent the items represent the construct to be measured (here self-efficacy) reported by the sample, and to what extent the sample represents the different levels on the continuum of the construct covered by the items. Good targeting is elemental for measurement precision and quality evaluation of the instrument. One indicator for assessment of targeting is the mean person location to be ± 0.5 from the mean item location (0 logits) [38]. A higher mean value indicates that the sample as a whole has higher self-efficacy than the average of the scale, and a lower mean value indicate that the sample as a whole has lower self-efficacy than the average of the scale [49].

#### Reliability

The capability of the instrument to distinguish persons into distinct groups can be evaluated through Person Separation Index (PSI), which is equivalent to Cronbach’s alpha. The minimum PSI value required for group use is 0.70 and 0.85 for individual use [49]. PSI can further be used to determine the strata, i.e. the number of statistically distinct groups of persons that can be differentiated by the instrument, separated by ≥ 3 standard errors [50, 51]. Higher PSI values indicate greater detection of reliable differences between persons.

#### Response category functioning

Whether the response categories function as intended, for example from less to more, can be assessed through response category functioning. Response category threshold is the location where there is an equal probability that a person answers in either of two adjacent response categories. Disordered thresholds occur if respondents are not able to differentiate between two response categories and imply that the response categories do not work as intended [40].

#### Rasch model fit

Sufficient model fit indicates that basic assumptions of local independency and unidimensionality are fulfilled and is critical for evaluation of measurement properties. There are different approaches to investigate to what extent the data fit into what is expected by the Rasch model. It can be statistically and graphically investigated. Statistically by standardized item fit residuals and Chi-squared statistics for individual items, and graphically through item characteristic curves (ICC) [37]. Respondents are grouped into class intervals according to their location on the logit scale. Standardized item fit residuals show the difference between the observed item responses and the model’s expected item responses in the respective class intervals. The expected fit residual value is 0, which means perfect fit, but the range ± 2.5 is considered acceptable. Low negative values indicate local dependency and high positive values indicate multidimensionality [49]. ICC is a graphical presentation of the difference between observed and expected responses. Chi-squared statistics for individual items further define the difference between observed and expected responses, the Chi-squares should not be significant to support model fit [37].

#### Differential item functioning

Differential item functioning (DIF) is an additional aspect of model fit and evaluates if items work in a similar way in different subgroups of respondents, e.g. age and gender. DIF is tested by a two-way ANOVA of the residuals across the levels of the construct being measured (here self-efficacy), in subgroups of respondents with similar scores [52]. DIF occurs when the subgroups respond differently to an item despite having the same level of the construct [37]. Uniform DIF means that there is a systematic difference in response probability between the subgroups across the levels of the construct. Non-uniform DIF means that the difference between the subgroups vary across the levels of the construct [49]. DIF was tested for age (< 25 vs. 25 +), gender (men and women), education level (previous university degree and lower education level) and previous health care education (yes and no). Year of birth was divided according to median, and gender was changed from the categories: “man”, “woman” and “other” into “men” and “women” because no respondent in the sample answered “other”.

#### Raw-score transformation to interval measurements

To facilitate use of total sum scores of the instrument in practice and research, raw score transformations to interval measurements can be performed. Since the raw scores are ordinal in nature, one point on the various items is not necessarily the same across the measurement spectrum. Through RUMM2030 the raw scores can be translated into logits. Given an appropriate solution, the Rasch person estimates in logits can be transformed into interval measurements of the same range as the original raw scores [53].

## Results

### Part 1

When the development part of the study was finalized, the instrument consisted of 20 + 20 items, 20 items about self-efficacy in knowledge- and 20 items about self-efficacy in ability to counsel persons about their lifestyle, hereafter labeled SELC 20 + 20 (see appendix 1). The results from the field-testing of the instrument showed that 96% (*n* = 23) found the instructions and items easy to understand, 100% (*n* = 24) found the items relevant and the average time to answer was 5 minutes.

### Part 2

Only those with answers on all items were included due to three reasons. First, number of persons with missing data in some items were few (9,7%). Second, the estimates provided from the Rasch model analysis becomes more stable, have less error. Third, some estimates can only be calculated with no missing responses, such as the Cronbach´s alpha and raw score transformations can only be done on complete data. See item response rates in Table 1.

**Table 1:** Item response rates for the SELC 20 + 20 (items 1–40) among nursing students (*n* = 310)

		Response category endorsement, %				
		*I am very insecure in my…*	*I am insecure in my…*	*I am sure of my…*	*I am very sure of my…*	Missing
**Knowledge**						
		*…confidence in theoretical knowledge about…*				
*Tobacco*						
i1	Identification of tobacco use	2	11	52	34	1
i2	Health effects of tobacco use	1	6	46	47	0
i3	Assessment of motivation for tobacco cessation	3	31	46	18	1
i4	Advice about tobacco	5	23	47	23	1
i5	Motivational strategies for tobacco cessation	1	36	43	13	2
*Alcohol*						
i6	Identification of alcohol consumption	3	13	52	30	1
i7	Health effects of alcohol consumption	1	9	50	41	0
i8	Assessment of motivation for decreased alcohol consumption	3	28	56	11	2
i9	Advice about alcohol	5	28	44	21	1
i10	Motivational strategies for decreased alcohol consumption	6	35	41	17	1
*Physical activity*						
i11	Identification of physical activity	1	6	42	50	1
i12	Health effects of physical activity	1	3	31	65	0
i13	Assessment of motivation for physical activity	1	16	55	27	0
i14	Advice about physical activity	1	10	42	46	1
i15	Motivational strategies for physical activity	1	17	47	34	1
*Eating habits*						
i16	Identification of eating habits	3	14	50	32	1
i17	Health effects of eating habits	1	5	48	46	0
i18	Assessment of motivation for healthier eating habits	16	23	54	20	1
i19	Advice about eating habits	2	13	48	36	0
i20	Motivational strategies for healthy eating habits	3	24	46	27	0
**Ability**						
		*… confidence in practical ability to…*				
*Tobacco*						
i21	Identification of tobacco use	3	19	50	27	1
i22	Health effects of tobacco use	1	14	52	31	3
i23	Assessment of motivation for tobacco cessation	6	35	42	14	2
i24	Advice about tobacco	5	29	44	20	2
i25	Motivational strategies for tobacco cessation	9	38	40	11	2
*Alcohol*						
i26	Identification of alcohol consumption	4	18	52	24	2
i27	Health effects of alcohol consumption	1	18	49	30	2
i28	Assessment of motivation for decreased alcohol consumption	4	35	47	11	2
i39	Advice about alcohol	6	36	39	17	2
i30	Motivational strategies for decreased alcohol consumption	10	38	36	14	2
*Physical activity*						
i31	Identification of physical activity	2	10	43	44	1
i32	Health effects of physical activity	1	5	41	51	1
i33	Assessment of motivation for physical activity	2	21	49	26	1
i34	Advice about physical activity	2	12	48	36	1
i35	Motivational strategies for physical activity	3	20	42	32	2
*Eating habits*						
i36	Identification of eating habits	3	19	50	27	1
i37	Health effects of eating habits	2	13	50	35	1
i38	Assessment of motivation for healthier eating habits	3	29	50	16	2
i39	Advice about eating habits	32	16	52	28	1
i40	Motivational strategies for healthy eating habits	5	29	44	21	1

#### Dimensionality and local dependency

To determine the different subconstructs, independent t-tests analyses were done. The analyses showed that tobacco use and alcohol consumption should be considered unidimensional but physical activity and eating habits need to be considered as separate subconstructs. Therefore, the two constructs knowledge and ability were divided into their 3 respective subconstructs (tobacco and alcohol; physical activity; eating habits).

A subtest analysis of the knowledge construct with the 3 subconstructs showed a 0.132 (C^2^) variance unique to the subscale, a 0.883 (r) latent correlation between the subscales and a non-error variance of 0.897 (A). The PSI (alpha) decreased from 0.906 (0.917) (all 20 items) to 0.812 (0.776) (subtests) due to local dependency in the item set. A subtest analysis of the ability construct with the 3 subconstructs showed a 0.099 (C^2^) variance unique to the subscale, a 0.910 (r) latent correlation between the subscales and a non-error variance of 0.907 (A). The PSI (alpha) decreased from 0.929 (0.937) (all 20 items) to 0.843 (0.803) (subtests) due to local dependency in the item set. The results of the subtest analyses support unidimensionality within the knowledge and ability constructs respectively, since the values for A and r were high, and the values for C^2^ were low. This justifies the use of total scores for each of the two constructs, separately.

**Fig. 2:**
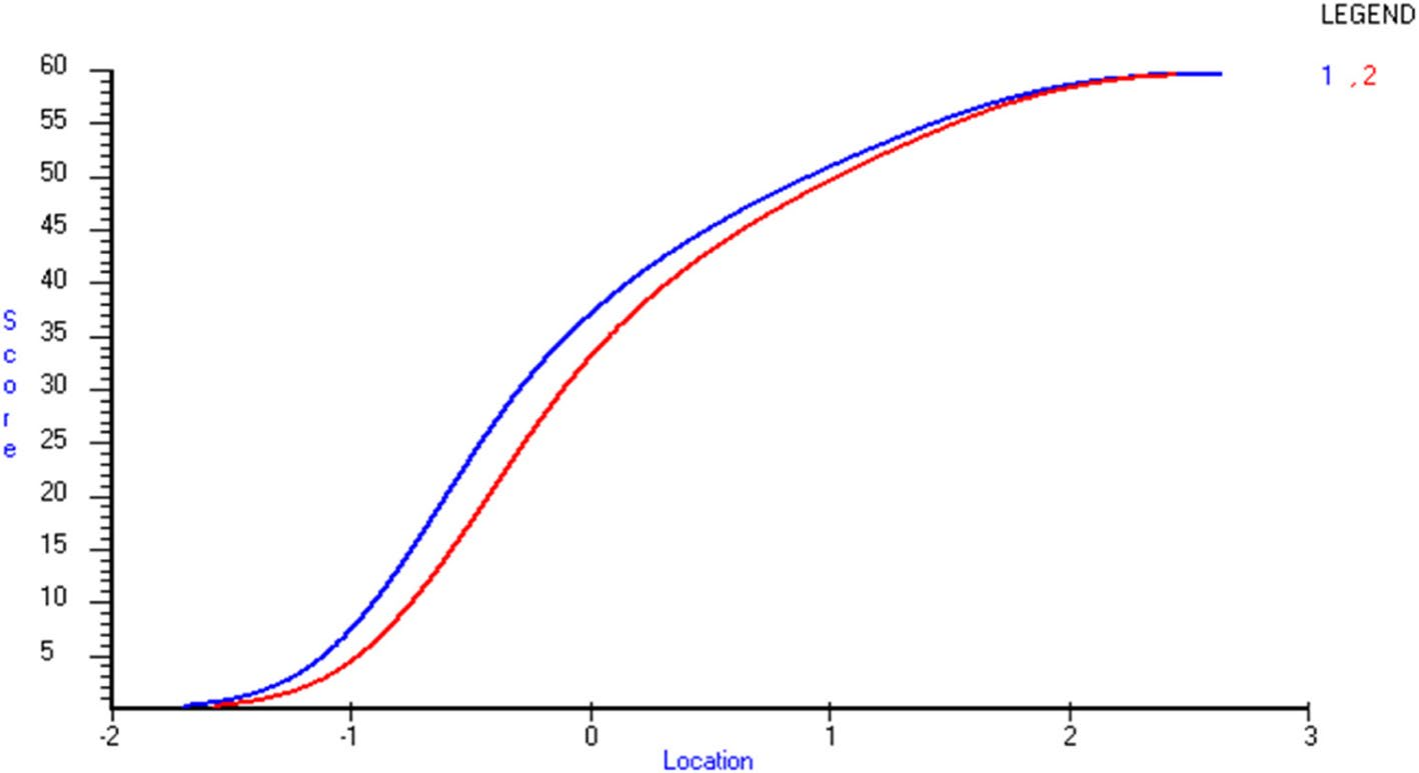
Independent t-test of Knowledge (1) and Ability (2). Representing logit location (x-axis) and score (y-axis)

Further on, an independent t-test of the 2 overall constructs (knowledge and ability) showed that respondents had a generally higher score on the knowledge construct compared to the ability construct (Fig. 2), indicating that knowledge is a prerequisite for ability.

Item residual correlations were investigated, first on the overall instrument with 40 items, which showed many (*n* = 81) instances of residual correlations over the Yen’s critical value (CV = 0.22) When knowledge and ability were analysed separately there were still many residual correlations, in the knowledge construct (CV > 0.17, *n* = 22) as well as in the ability construct (CV > 0.17, *n* = 23). To resolve local dependency, and due to the findings from the dimensionality analyses (described above), the two constructs of the instrument (knowledge and ability) were divided into 3 respective subconstructs: tobacco and alcohol; physical activity; and eating habits. Within the knowledge construct there was still a tendency to local dependence (CV > 0.17) within the “tobacco and alcohol” subconstruct (Q3,_*_ = maximum residual correlation (r) 0.308 – mean *r* -0.109 = 0.417) while this was less distinct regarding “physical activity” (Q3,_*_ = 0.010—-0.241 = 0.251) and “eating habits” (Q3,_*_ = 0.006- -0.245 = 0.251). A similar pattern was found within the ability construct, tending to local dependence within “tobacco and alcohol” subconstruct (Q3,_*_ = 0.218—-0.110 = 0.328) while this was less distinct regarding “physical activity” (Q3,_*_ = 0.021—-0.241 = 0.262) and “eating habits” (Q3,_*_ = 0.017—-0.246 = 0.263). If considering the more liberal rule where residual correlations over 0.3 are considered to indicate local dependency, there was only one case of local dependency. This was within the knowledge construct, within the “tobacco and alcohol” subconstruct, between items 2 (health effects of tobacco use) and 7 (health effects of alcohol consumption), *r* = 0.308.

#### Targeting

**Fig. 3:**
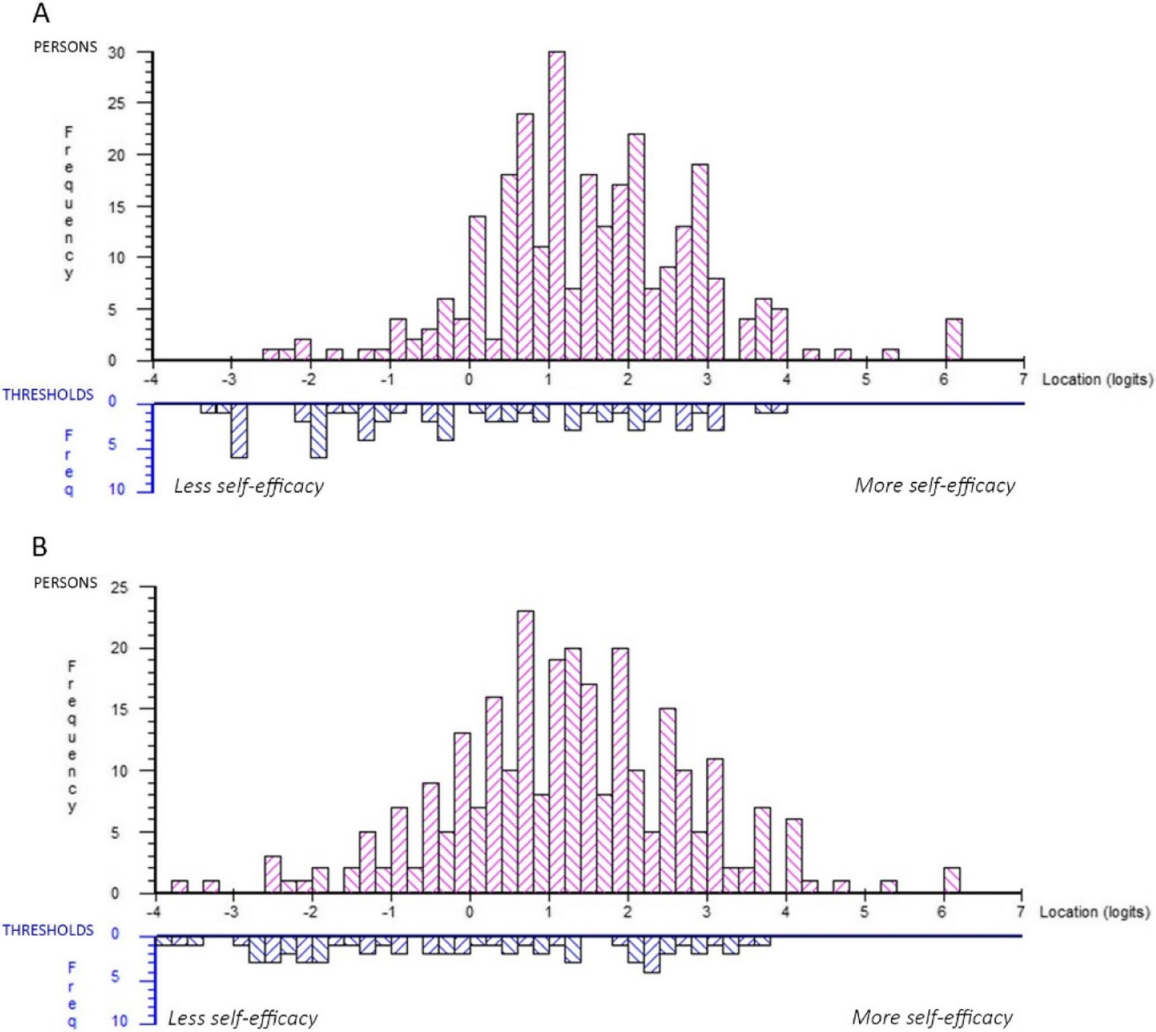
Person item threshold distribution, distribution of respondents (upper panels) and response category thresholds (lower panels) on the common logit metric from less to more self-efficacy (x-axis). In panel **A** for Knowledge and **B** for Ability

The 2 constructs of the instrument: knowledge and ability, with their 3 respective subconstructs represent a quantitative continuum from less to more self-efficacy. The knowledge construct ranged from approximately –3.4 to 4 logits, with a gap between –2.2 to –2.8, and the ability construct ranged from approximately –4 to 3.8 logits, with no major gaps. In both constructs, there were a small ceiling effect, meaning that items do not represent respondents at the highest levels of self-efficacy (Fig. 3). The mean person location relative to the items was for the knowledge construct 1.554 (SD 1.360) and for the ability construct 1.240 (SD 1.526). This means that the items represent a lower level of self-efficacy than that reported by the sample.

#### Reliability

As mentioned, the reliability for the knowledge construct was PSI 0.812 and for the ability construct 0.843. The reliabilities implies that 3 distinct levels of self-efficacy could be identified [51], both from the knowledge as well as the ability constructs.

#### Response category functioning

**Fig. 4:**
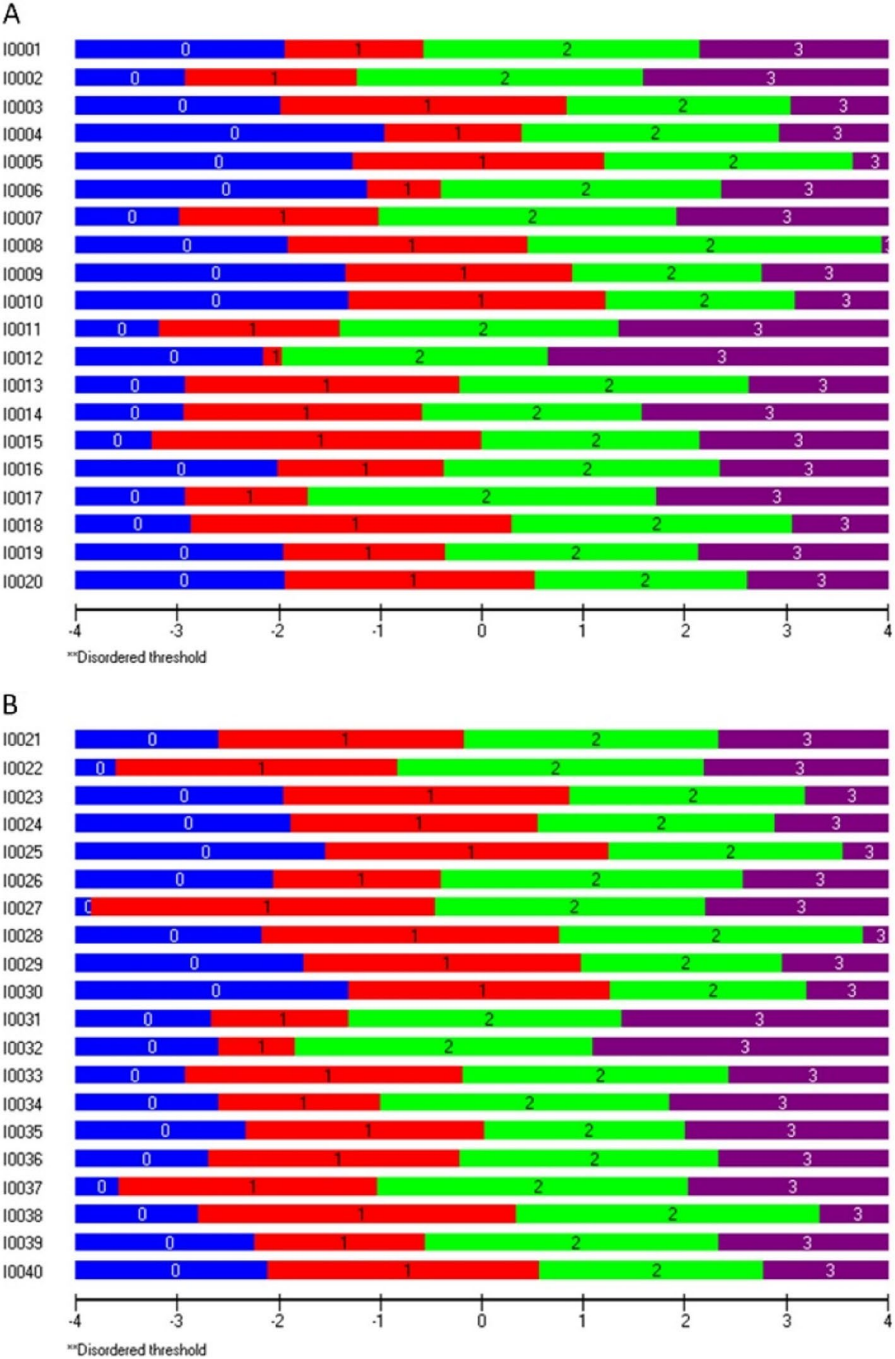
Response category functioning of the SELC 20 + 20. Areas 0–3 correspond with the 4 response categories (0 = I am very insecure in my…, 1 = I am insecure in my…, 2 = I am sure of my…, 3 = I am very sure of my…). In panel A for Knowledge and B for Ability

The response categories functioned as intended, from less to more self-efficacy, without any disordered thresholds (Fig. 4).

#### Rasch model fit

**Fig. 5:**
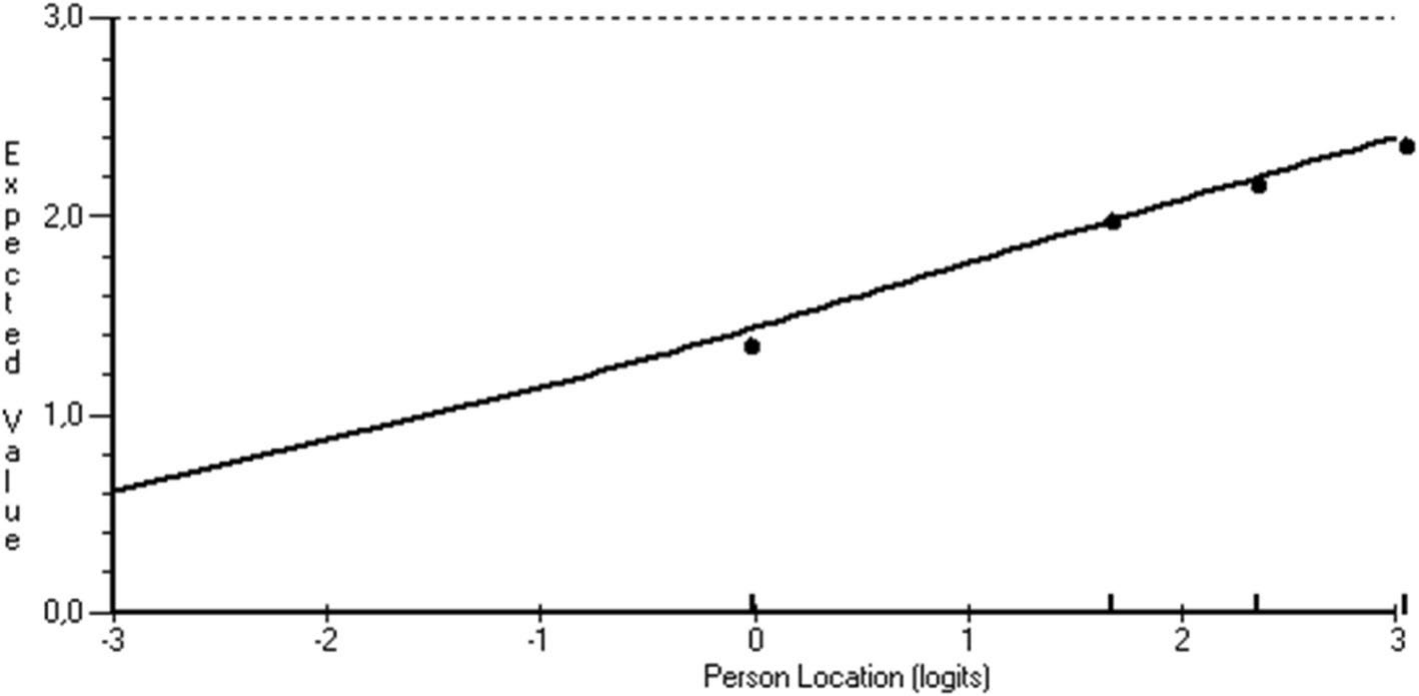
Item characteristic curve (ICC), representing expected item responses (y-axis) from less to more self-efficacy (x-axis) for item 14 (“Advice about physical activity”). Black dots are item responses by subgroups of respondents with various levels of self-efficacy and the line is the expected ICC

On the item level, one item (i14) had a significant fit residual outside the accepted range of ± 2.5 (-2.964) (Table 2). Although, the ICC of i14 was acceptable (Fig. 5). Besides this item also another item (i32) showed a significant deviation from the Rasch model, but the fit residual (-0.721) was within the acceptable range (-2.5 – 2.5). In addition, when adjusting the sample size to *n* = 200 (which can be done in RUMM2030, since the chi-square statistics are sensitive to large samples, the p-values were no longer significant (for i14 and i32). On the person level, in the knowledge construct 28 (10%) respondents had fit residuals below –2.5 and 5 (1.8%) respondents above 2.5. Regarding the ability construct, 34 (12.1%) respondents had fit residuals below –2.5 and 8 (2.8%) respondents above 2.5.

**Table 2:** SELC 20 + 20 item level Rasch model location and fit statistics (complete cases only, *n* = 280). Sorted according to location within each subconstruct (from less to more self-efficacy)

*Subconstructs* and items		Location (SE)	Fit Residual	Chi-square	*P*-value
**Knowledge**					
*Tobacco and alcohol*					
i2	Health effects of tobacco use	-1.192 (0.113)	0.050	1.506	0.826
i7	Health effects of alcohol consumption	-1.039 (0.111)	0.357	6.154	0.188
i1	Identification of tobacco use	-0.527 (0.104)	0.906	2.541	0.637
i6	Identification of alcohol consumption	-0.123 (0.100)	0.565	1.631	0.803
i3	Assessment of motivation for tobacco cessation	0.234 (0.099)	1.275	7.481	0.112
i9	Advice about alcohol	0.371 (0.093)	-0.749	1.352	0.852
i4	Advice about tobacco	0.393 (0.095)	-1.891	4.697	0.320
i8	Assessment of motivation for decreased alcohol consumption	0.444 (0.110)	0.310	4.953	0.292
i10	Motivational strategies for decreased alcohol consumption	0.616 (0.095)	-0.775	2.574	0.631
i5	Motivational strategies for tobacco cessation	0.824 (0.100)	-0.149	5.996	0.199
*Physical activity*					
i12	Health effects of physical activity	-0.912 (0.141)	0.854	8.602	0.072
i11	Identification of physical activity	-0.579 (0.133)	1.388	1.385	0.847
i14	Advice about physical activity	0.043 (0.122)	-2.964	15.156	0.004
i15	Motivational strategies for physical activity	0.613 (0.118)	0.310	2.465	0.651
i13	Assessment of motivation for physical activity	0.835 (0.125)	0.365	2.133	0.711
*Eating habits*					
i17	Health effects of eating habits	-1.294 (0.133)	-0.298	8.218	0.084
i19	Advice about eating habits	0.003 (0.116)	-1.301	6.656	0.155
i16	Identification of eating habits	0.158 (0.118)	0.507	2.013	0.733
i18	Assessment of motivation for healthier eating habits	0.431 (0.124)	0.914	3.347	0.501
i20	Motivational strategies for healthy eating habits	0.703 (0.112)	-1.042	2.847	0.584
**Knowledge**					
*Tobacco and alcohol*					
i27	Health effects of alcohol consumption	-1.164 (0.110)	-0.725	3.339	0.503
i22	Health effects of tobacco use	-1.114 (0.112)	-0.497	2.561	0.633
i26	Identification of alcohol consumption	-0.282 (0.105)	0.785	3.540	0.471
i24	Advice about tobacco	0.196 (0.100)	-0.851	3.809	0.432
i39	Advice about alcohol	0.407 (0.098)	-1.744	2.416	0.660
i23	Assessment of motivation for tobacco cessation	0.410 (0.101)	0.569	7.489	0.112
i28	Assessment of motivation for decreased alcohol consumption	0.446 (0.106)	0.564	7.652	0.105
i21	Identification of tobacco use	0.470 (0.103)	1.784	8.818	0.066
i30	Motivational strategies for decreased alcohol consumption	0.771 (0.097)	-1.673	3.269	0.513
i25	Motivational strategies for tobacco cessation	0.800 (0.101)	0.109	5.512	0.239
*Physical activity*					
i32	Health effects of physical activity	-1.051 (0.136)	-0.721	14.712	0.005
i31	Identification of physical activity	-0.442 (0.128)	1.425	5.747	0.219
i34	Advice about physical activity	0.054 (0.127)	-2.183	6.941	0.139
i33	Assessment of motivation for physical activity	0.622 (0.123)	0.796	8.712	0.082
i35	Motivational strategies for physical activity	0.817 (0.115)	-0.876	7.469	0.113
*Eating habits*					
i37	Health effects of eating habits	-1.210 (0.128)	-0.662	8.961	0.062
i39	Advice about eating habits	-0.097 (0.119)	-1.042	7.023	0.135
i36	Identification of eating habits	-0.073 (0.119)	1.307	4.629	0.327
i38	Assessment of motivation for healthier eating habits	0.617 (0.125)	-1.051	7.843	0.097
i40	Motivational strategies for healthy eating habits	0.764 (0.113)	-1.331	7.582	0.108

#### Differential Item Functioning

There were no uniform or non-uniform DIF for any items regarding age, gender, education level or previous health care education, neither in the knowledge nor in the ability constructs.

#### Raw score transformation to interval measurements

The final version of the instrument consisted of 20 items about self-efficacy in knowledge- and 20 items about self-efficacy in ability to counsel persons about their lifestyle. In the final version of the instrument, we revised the coding of item responses from 1–4 to 0–3. Thus, the total score within each construct (knowledge and ability) ranged from 0–60. In the appendix, raw scores of the instrument’s constructs knowledge and ability were transformed to linear logit values (together with their standard errors) and to linearized scores using an online tool [53] (see appendix 2).

## Discussion

This study describes the development and quality assessment of an instrument measuring self-efficacy in lifestyle counselling. In Sweden, it has been shown that many patients do not receive lifestyle counselling even if it is regulated in national guidelines [12, 54]. Self-efficacy has been shown to positively predict the engagement in lifestyle counselling [55] which in turn provides an opportunity to promote health and prevent disease. As there was no instrument measuring self-efficacy in lifestyle counselling available in Swedish, an instrument was needed. The instrument was inspired by an existing instrument, and developed according to the recommendations for constructing self-efficacy instruments described by Bandura [23]. Bandura advocates for the use of all three dimensions of self-efficacy in instruments to measure self-efficacy: *magnitude, strength, and generality* [25]. *Magnitude* measures the perceived level of difficulty, *strength* how confident the respondent is, and *generality* if and how self-efficacy beliefs are positively related across domains or time [26]. By using a Likert-scale *magnitude* and *strength* is measured simultaneously [56], including both knowledge and ability further enhance the magnitude dimension because ability has been shown to be perceived as a harder skill than knowledge in a previous study [57]. The subtest analyses in this study confirmed that by showing a generally higher score on the knowledge construct compared to the ability construct, indicating that ability is more demanding than knowledge and that knowledge may be a prerequisite for ability (Fig. 2). Despite the above reasoning, Bandura recommends a 0–100 response format for measuring self-efficacy [27]. However, we choose to use a 4-point semantically anchored Likert-scale to increase usability [31]. A comparison of the 0–100 response format to a Likert-scale has shown equally high reliability [56].

During the psychometric analyses, the instrument was named SELC 20 + 20. SELC 20 + 20 does not include a test of theoretical knowledge, which has been used in other studies to compare knowledge and self-assessed ability [57, 58]. In SELC 20 + 20 both knowledge and ability are self-assessed and therefore subjective measures, which might be one explanation of the ceiling effect and high mean person location relative to the items seen in the analysis (Table 1 and Fig. 3). As a comparison, Stump et al. (2012) developed an instrument to measure nursing students’ self-efficacy in care for critically ill patients, this instrument also received very high estimates of self-efficacy and the authors argue that students might be uncomfortable to admit low levels of self-efficacy [59]. The researchers in the present study gave the respondents information that the instrument was completely anonymous and, to avoid the same result as Stump et al. (2012), that their participation in the study would in no way affect their grades, this was also important from an ethical perspective. However, the instrument aims to measure self-efficacy in lifestyle counselling, which is not necessarily dependent on actual knowledge and ability but rather on a person's capability to do the best they can with whatever skills they have in a given situation [23]. Schunk and Pajares (2009) even argue that the optimal level of self-efficacy is slightly higher than the level of knowledge or ability, because it creates a positive feedback loop between confidence and learning [29].

Concerning *generality*, van der Bijl et al. (2001) gives an example of how between-domain generality can be evaluated in a self-efficacy instrument regarding diabetes, by including items about perceived self-efficacy to control blood sugar and perceived self-efficacy to inject insulin [60]. Related to the results of this present study, inclusion of the four lifestyle habits: tobacco, alcohol, physical activity and eating habits, covers the domains of lifestyle counselling in Sweden. Through including all domains, generality of lifestyle counselling self-efficacy is measured in the SELC 20 + 20 in accordance with Bandura (1982) but from a Swedish perspective [61]. In summary, the SELC 20 + 20 can be considered to measure all three domains of self-efficacy recommended by Bandura [25].

For the cognitive interviews during the development part of the study, both nursing students, university teachers within health sciences and clinical experts working with national guidelines for disease prevention in the healthcare sector were included. This gave a variety of opinions regarding language and relevance of items, which was a strength. The interviews were conducted by three different researchers (SA, MR, LB), minimum two researchers listened to each interview and three researchers discussed the findings before each revision of the instrument.

The sample for the quality assessment were nursing students at a university in the south of Sweden. The results might have been different with other population groups, e.g. clinical nurses or nursing students in other cultural contexts. Test–retest was not done in this study but needs to be tested in the future to evaluate the SELC 20 + 20’s stability over time.

The SELC 20 + 20 was found to be easy to understand with an acceptable response burden. Despite some minor misfit and local dependency, we decided to maintain all items due to three reasons. First, since all items are relevant to assess and second, to maintain the same logic throughout the instrument. Thirdly, when the total score of an instrument consists of subscales, some local dependency within the same subscale is expected [37].

The analyses confirmed that knowledge and ability are two different, but related, constructs. Further on, the analyses showed that tobacco use, and alcohol consumption could be summed together but physical activity and eating habits need to be summed separately. Therefore, the two constructs knowledge and ability were divided into three respective subconstructs: tobacco and alcohol, physical activity as well as eating habits, were the result of each subconstruct can be summed to assess self-efficacy in lifestyle counselling. Each subconstruct provides unique information. However, it is also possible to sum the score within each of the knowledge and the ability constructs, as indicated by the indices from the subtest analyses.

The SELC 20 + 20 instrument can hopefully be a usable tool in the professional educations of health care personnel, e.g. nurses, assistant nurses, medical students, paramedics and public health practitioners, as well as clinically in all areas of healthcare, in the future. Both to assess if there is a need for training in knowledge and/or ability in lifestyle counselling and to evaluate if a course or training increase self-efficacy. The findings of this study indicate good measurement properties of SELC 20 + 20 through unidimensionality, manageable local dependency, good reliability for group use, ordered thresholds, acceptable rasch model fit, and no DIF. Future studies are needed to evaluate stability (test–retest), as well as the psychometric properties of the SELC 20 + 20 instrument in other population groups, languages and cultures, as well as evaluate if there is any association between learning methods and self-efficacy in knowledge and ability in lifestyle counselling.

## Conclusion

RMT was considered the most complete method to rate this clinimetric scale [34, 35]. SELC 20 + 20 was found to be easy to understand with an acceptable respondent burden and the instrument showed good measurement properties. The analyses showed that knowledge and ability are two different, but related, constructs, where ability is more demanding than knowledge. The constructs knowledge and ability can be summed into three respective related subconstructs: tobacco and alcohol, physical activity as well as eating habits, to assess self-efficacy in lifestyle counselling. In addition, all 20 items within the knowledge construct as well as the 20 items within ability construct can be summed, achieving two separate but related total scores, where knowledge is a prerequisite for ability. The long-term goal with this instrument is to facilitate the evaluation of healthcare professionals’ lifestyle counselling self-efficacy. Evaluation can enable an implementation of interventions to increase it, when necessary, which could possibly lead to a higher percentage of patients receiving lifestyle counselling in the future. A lot of research and clinical work is needed to reach the long-term goal. Although, this study indicates that SELC 20 + 20 can be a useful tool in nursing educations. Conceivably both to better understand students’ general lifestyle counselling self-efficacy, as well as to evaluate university courses that aim to increase it. However, future studies need to evaluate this further. The promising results from this study opens up many clinimetric research possibilities. Initially, the instrument needs to be tested with different target populations, e.g. other healthcare educations (assistant nurses, medical doctors, paramedics and public health practitioners) as well as with clinically working healthcare professionals among all professions listed above.

### Supplementary Information

**Additional file 1:** Self-Efficacy in Lifestyle Counselling scale – SELC 20 + 20.

**Additional file 2: **Appendix. Translation of raw scores. score locations to linearized scores.

## Data Availability

The data are available from the corresponding author on reasonable request.

## Abbreviations

NCD: Non-communicable diseases
HPCSES: Health Promotion Counselling Self-Efficacy Scale
CTT: Classical Test Theory
MTT: Modern Test Theory
RMT: Rasch Measurement Theory
DIF: Differential Item Functioning
PSI: Person Separation Index
ICC: Item Characteristic Curves
SELC: Self-Efficacy in Lifestyle Counselling scale
CV: Critical Value
SD: Standard Deviation

## References

[1] Knapper JT, Ghasemzadeh N, Khayata M, Patel SP, Quyyumi AA, Mendis S (2015). Time to Change Our Focus: Defining, Promoting, and Impacting Cardiovascular Population Health. J Am Coll Cardiol.

[2] World Health Organization. The updated Appendix 3 of the WHO Global NCD Action Plan 2013–2020 [Internet]. Switzerland: World Health Organisation; 2017. [cited 2023 April 20]. Available from: https://iris.who.int/bitstream/handle/10665/330805/9789240000490-eng.pdf?sequence=1.

[3] World Health Organization. Noncommunicable diseases progress monitor 2020. Geneva: World Health Organization; 2020. [cited 2023 April 20]. Available from: https://iris.who.int/bitstream/handle/10665/330805/9789240000490-eng.pdf?sequence=1.

[4] GBD 2019 Diseases and Injuries Collaborators. Global burden of 369 diseases and injuries in 204 countries and territories, 1990–2019: a systematic analysis for the Global Burden of Disease Study 2019. Lancet. 2020;396(10258):1204–22. 10.1016/S0140-6736(20)30925-9 PMC7567026 33069326

[5] Collaborators GRF (2018). Global, regional, and national comparative risk assessment of 84 behavioural, environmental and occupational, and metabolic risks or clusters of risks for 195 countries and territories, 1990–2017: a systematic analysis for the Global Burden of Disease Study 2017. Lancet.

[6] May AM, Struijk EA, Fransen HP, Onland-Moret NC, de Wit GA, Boer JM (2015). The impact of a healthy lifestyle on Disability-Adjusted Life Years: a prospective cohort study. BMC Med.

[7] Yusuf S, Hawken S, Ounpuu S, Dans T, Avezum A, Lanas F (2004). Effect of potentially modifiable risk factors associated with myocardial infarction in 52 countries (the INTERHEART study): case-control study. Lancet.

[8] World Health Organization. Global action plan for the prevention and control of noncommunicable diseases 2013–2020 [Internet]. Geneva: World Health Organisation; 2013. [cited 2023 April 22]. Available from: https://iris.who.int/bitstream/handle/10665/94384/9789241506236_eng.pdf?sequence=1&isAllowed=y.

[9] Hälso- och sjukvårdslag (SFS 1982:763). Stockholm: Socialdepartementet. [cited 2023 April 22]. Available from: https://www.riksdagen.se/sv/dokument-och-lagar/dokument/svensk-forfattningssamling/halso--och-sjukvardslag-1982763_sfs-1982-763/.

[10] Socialstyrelsen. Så här vill patienter berätta för sjukvården om sina levnadsvanor, resultat av en befolkningsundersökning 2016. Stockholm: Socialstyrelsen; 2016. [cited 2023 April 22]. Available from: https://www.socialstyrelsen.se/globalassets/sharepoint-dokument/artikelkatalog/ovrigt/2016-12-13.pdf.

[11] Sveriges Kommuner och Regioner. Hälso- och sjukvårdsbarometern 2019. Stockholm: Sveriges Kommuner och Regioner; 2019. [cited 2023 April 22]. Available from: https://skr.se/download/18.7c1c4ddb17e3d28cf9b61643/1642599970977/7585-875-3.pdf.

[12] Brobeck E, Bergh H, Odencrants S, Hildingh C (2015). Lifestyle advice and lifestyle change: to what degree does lifestyle advice of healthcare professionals reach the population, focusing on gender, age and education?. Scand J Caring Sci.

[13] Socialstyrelsen. Nationella riktlinjer för prevention och behandling vid ohälsosamma levnadsvanor. Sverige: Socialstyrelsen; 2018. [cited 2023 April 22]. Available from: https://www.socialstyrelsen.se/globalassets/sharepoint-dokument/artikelkatalog/nationella-riktlinjer/2018-6-24.pdf.

[14] Ampt AJ, Amoroso C, Harris MF, McKenzie SH, Rose VK, Taggart JR (2009). Attitudes, norms and controls influencing lifestyle risk factor management in general practice. BMC Fam Pract.

[15] Brobeck E, Bergh H, Odencrants S, Hildingh C (2011). Primary healthcare nurses' experiences with motivational interviewing in health promotion practice. J Clin Nurs.

[16] Hörnsten Å, Lindahl K, Persson K, Edvardsson K (2014). Strategies in health-promoting dialogues–primary healthcare nurses' perspectives–a qualitative study. Scand J Caring Sci.

[17] Jallinoja P, Absetz P, Kuronen R, Nissinen A, Talja M, Uutela A (2007). The dilemma of patient responsibility for lifestyle change: perceptions among primary care physicians and nurses. Scand J Prim Health Care.

[18] James S, Halcomb E, Desborough J, McInnes S (2019). Lifestyle risk communication by general practice nurses: An integrative literature review. Collegian.

[19] Jansink R, Braspenning J, van der Weijden T, Elwyn G, Grol R (2010). Primary care nurses struggle with lifestyle counseling in diabetes care: a qualitative analysis. BMC Fam Pract.

[20] Keyworth C, Epton T, Goldthorpe J, Calam R, Armitage CJ (2019). 'It's difficult, I think it's complicated': Health care professionals' barriers and enablers to providing opportunistic behaviour change interventions during routine medical consultations. Br J Health Psychol.

[21] Lambe B, Connolly C, McEvoy R (2008). The determinants of lifestyle counselling among practice nurses in Ireland. Int J Health Promot Educ.

[22] Röing M, Hederberg M, Holmström IK (2014). (Tele) Health Promotion in Primary Healthcare Centers—An Exploratory Study. Vård i Norden.

[23] Bandura A (1997). Self-efficacy: The exercise of control.

[24] Bandura A (2001). Social cognitive theory: an agentic perspective. Annu Rev Psychol.

[25] Bandura A. Social foundations of thought and action: a social cognitive theory', in’Prentice Hall Series in Social Learning theory’. 2007;9780138156145.

[26] Bandura A (1977). Self-efficacy: Toward a unifying theory of behavioral change. Psychol Rev.

[27] Urdan T, Pajares F, editors. Self-Efficacy Beliefs of Adolescents. 1st ed. IAP: Greenwich; 2006.

[28] Laschinger HK, Tresolini CP (1999). An exploratory study of nursing and medical students health promotion counselling self-efficacy. Nurse Educ Today.

[29] Schunk DH, Pajares F (2009). Self-Efficacy Theory.

[30] Willis GB. Cognitive interviewing: A tool for improving questionnaire design. 1st ed. Sage Publications Inc; 2004.

[31] Wenemark M (2017). Enkätmetodik med respondenten i fokus.

[32] Stenner A, Stone M, Fisher JW (2018). The unreasonable effectiveness of theory based instrument calibration in the natural sciences: What can the social sciences learn?. J Phys: Conf Ser.

[33] Carrozzino D, Patierno C, Guidi J, Berrocal Montiel C, Cao J, Charlson ME (2021). Clinimetric Criteria for Patient-Reported Outcome Measures. Psychother Psychosom.

[34] Carrozzino D, Patierno C, Pignolo C, Christensen KS (2023). The concept of psychological distress and its assessment: A clinimetric analysis of the SCL-90-R. Int J Stress Manag.

[35] Christensen KS, Cosci F, Carrozzino D, Sensky T. Rasch Analysis and Its Relevance to Psychosomatic Medicine. Psychother Psychosom. 2024;1–6. Epub 2024 Jan 4. 10.1159/000535633. 10.1159/000535633 38176396

[36] Rasch G. Probabilistic Models for Some Intelligence and Attainment Tests. 1st ed. Chicago: MESA Press; 1993.

[37] Andrich D, Marais I. A course in Rasch measurement theory. 1st ed. Singapore: Springer Verlag; 2019.

[38] Hagquist C, Bruce M, Gustavsson JP (2009). Using the Rasch model in nursing research: an introduction and illustrative example. Int J Nurs Stud.

[39] Hagell P, Westergren A (2016). Sample Size and Statistical Conclusions from Tests of Fit to the Rasch Model According to the Rasch Unidimensional Measurement Model (Rumm) Program in Health Outcome Measurement. J Appl Meas.

[40] Hobart J, Cano S (2009). Improving the evaluation of therapeutic interventions in multiple sclerosis: the role of new psychometric methods. Health Technol Assess..

[41] Andrich DSB (2019). Rumm 2030: Rasch Unidimensional Measurement Models (software).

[42] Westergren A, Wictorin K, Hansson O, Hagell P (2022). Novel insights regarding the measurement properties of the SCOPA-AUT. BMC Neurol.

[43] Armstrong RA (2014). When to use the Bonferroni correction. Ophthalmic Physiol Opt.

[44] Smith EV (2002). Detecting and evaluating the impact of multidimensionality using item fit statistics and principal component analysis of residuals. J Appl Meas.

[45] Tennant A, Pallant JF (2006). Unidimensionality matters!(A tale of two Smiths?). Rasch measurement transactions.

[46] Marais I, Christensen KB, Kreiner S, Mesbah M (2013). Local Dependence. Rasch Models in Health.

[47] Christensen KB, Makransky G, Horton M (2017). Critical Values for Yen's Q(3): Identification of Local Dependence in the Rasch Model Using Residual Correlations. Appl Psychol Meas.

[48] Guttersrud Ø, Naigaga MD, Pettersen KS (2015). Measuring Maternal Health Literacy in Adolescents Attending Antenatal Care in Uganda: Exploring the Dimensionality of the Health Literacy Concept Studying a Composite Scale. J Nurs Meas.

[49] Tennant A, Conaghan PG (2007). The Rasch measurement model in rheumatology: what is it and why use it? When should it be applied, and what should one look for in a Rasch paper?. Arthritis Rheum.

[50] Wright BD, Masters GN (1982). Rating scale analysis.

[51] Schumacker RE, Smith EV (2007). A Rasch Perspective. Educ Psychol Measur.

[52] Hagquist C, Andrich D (2017). Recent advances in analysis of differential item functioning in health research using the Rasch model. Health Qual Life Outcomes.

[53] Ekstrand J, Westergren A, Årestedt K, Hellström A, Hagell P (2022). Transformation of Rasch model logits for enhanced interpretability. BMC Med Res Methodol.

[54] Kardakis T, Jerdén L, Nyström ME, Weinehall L, Johansson H (2018). Implementation of clinical practice guidelines on lifestyle interventions in Swedish primary healthcare - a two-year follow up. BMC Health Serv Res.

[55] Laschinger HK, McWilliam CL, Weston W (1999). The effects of family nursing and family medicine clinical rotations on nursing and medical students' self-efficacy for health promotion counseling. J Nurs Educ.

[56] Maurer TJ, Pierce HR (1998). A comparison of Likert scale and traditional measures of self-efficacy. J Appl Psychol.

[57] Tresolini CP, Stritter FT (1994). An analysis of learning experiences contributing to medical students' self-efficacy in conducting patient education for health promotion. Teach Learn Med.

[58] Silverplats J, Södersved Källestedt ML, Wagner P, Ravn-Fischer A, Äng B, Strömsöe A (2020). Theoretical knowledge and self-assessed ability to perform cardiopulmonary resuscitation: a survey among 3044 healthcare professionals in Sweden. Eur J Emerg Med.

[59] Stump GS, Husman J, Brem SK (2012). The Nursing Student Self-Efficacy Scale: Development Using Item Response Theory. Nurs Res..

[60] van der Bijl JJ, Shortridge-Baggett LM (2001). The Theory and Measurement of the Self-Efficacy Construct. Sch Inq Nurs Pract.

[61] Bandura A (1982). Self-efficacy mechanism in human agency. Am Psychol.

[62] World Medical Association. Declaration of Helsinki - Ethical Principles for Medical Research Involving Human Subjects. Helsinki. 1964 [updated 220906; cited 221110. Available from: https://www.wma.net/policies-post/wma-declaration-of-helsinki-ethical-principles-for-medical-research-involving-human-subjects/.

[63] Lag om etikprövning av forskning som avser människor (SFS 2003:460). Stockholm: Utbildningsdepartementet. [cited 2023 April 25]. Available from: https://www.riksdagen.se/sv/dokument-och-lagar/dokument/svensk-forfattningssamling/lag-2003460-om-etikprovning-av-forskning-som_sfs-2003-460/.

