# Supplementary material for: Development and quality assessment of the psychometric properties of the Self-Efficacy in Lifestyle Counselling scale (SELC 20 + 20) using Rasch analysis
Source: Health Qual Life Outcomes. 2024 Feb 23;22:20. doi: 10.1186/s12955-024-02236-z (PMC10885596; doi:10.1186/s12955-024-02236-z)
Supplement: Supplementary file 1 — Additional file 1: Self-Efficacy in Lifestyle Counselling scale – SELC 20 + 20. [file 12955_2024_2236_MOESM1_ESM.pdf]

# Self-Efficacy in Lifestyle Counselling scale – SELC 20 + 20

The lifestyle habits tobacco, alcohol, physical activity and eating habits are of great importance both for health promotion, as well as prevention and treatment of non-communicable diseases. The questionnaire is about self-efficacy in your knowledge and ability to counsel persons about their lifestyle.

| For each question, choose how confident you are in your theoretical **KNOWLEDGE** and practical **ABILITY** to counsel persons within the respective lifestyle habits. | **Circle <u>one</u> number based on the following answer options:**<br><br>1. I am very insecure in my…<br>2. I am insecure in my…<br>3. I am sure of my…<br>4. I am very sure of my… | | | | | | | |
|---|---|---|---|---|---|---|---|---|
| | … confidence in theoretical **knowledge** about/to… | | | | … confidence in practical **ability** about/to… | | | |
| **Tobacco** | | | | | | | | |
| 1. Identification of tobacco use | 1 | 2 | 3 | 4 | 1 | 2 | 3 | 4 |
| 2. Health effects of tobacco use | 1 | 2 | 3 | 4 | 1 | 2 | 3 | 4 |
| 3. Assessment of motivation for tobacco cessation | 1 | 2 | 3 | 4 | 1 | 2 | 3 | 4 |
| 4. Advice about tobacco | 1 | 2 | 3 | 4 | 1 | 2 | 3 | 4 |
| 5. Motivational strategies for tobacco cessation | 1 | 2 | 3 | 4 | 1 | 2 | 3 | 4 |
| **Alcohol** | | | | | | | | |
| 6. Identification of alcohol consumption | 1 | 2 | 3 | 4 | 1 | 2 | 3 | 4 |
| 7. Health effects of alcohol consumption | 1 | 2 | 3 | 4 | 1 | 2 | 3 | 4 |
| 8. Assessment of motivation for decreased alcohol consumption | 1 | 2 | 3 | 4 | 1 | 2 | 3 | 4 |
| 9. Advice about alcohol | 1 | 2 | 3 | 4 | 1 | 2 | 3 | 4 |
| 10. Motivational strategies for decreased alcohol consumption | 1 | 2 | 3 | 4 | 1 | 2 | 3 | 4 |
| **Physical activity** | | | | | | | | |
| 11. Identification of physical activity | 1 | 2 | 3 | 4 | 1 | 2 | 3 | 4 |
| 12. Health effects of physical activity | 1 | 2 | 3 | 4 | 1 | 2 | 3 | 4 |
| 13. Assessment of motivation for physical activity | 1 | 2 | 3 | 4 | 1 | 2 | 3 | 4 |
| 14. Advice about physical activity | 1 | 2 | 3 | 4 | 1 | 2 | 3 | 4 |
| 15. Motivational strategies for physical activity | 1 | 2 | 3 | 4 | 1 | 2 | 3 | 4 |
| **Eating habits** | | | | | | | | |
| 16. Identification of eating habits | 1 | 2 | 3 | 4 | 1 | 2 | 3 | 4 |
| 17. Health effects of eating habits | 1 | 2 | 3 | 4 | 1 | 2 | 3 | 4 |
| 18. Assessment of motivation for healthier eating habits | 1 | 2 | 3 | 4 | 1 | 2 | 3 | 4 |
| 19. Advice about eating habits | 1 | 2 | 3 | 4 | 1 | 2 | 3 | 4 |
| 20. Motivational strategies for healthy eating habits | 1 | 2 | 3 | 4 | 1 | 2 | 3 | 4 |

***Thank you for your participation!***

© 2024 Sara Alenius, Marie Rask, Petra Nilsson Lindström, Marie Nilsson, Lina Behm. The instrument may only be used in its entirety and with reference to the authors.
