# Supplementary material for: Development and quality assessment of the psychometric properties of the Self-Efficacy in Lifestyle Counselling scale (SELC 20 + 20) using Rasch analysis
Source: Health Qual Life Outcomes. 2024 Feb 23;22:20. doi: 10.1186/s12955-024-02236-z (PMC10885596; doi:10.1186/s12955-024-02236-z)
Supplement: Supplementary file 2 — Additional file 2: Appendix. Translation of raw scores. score locations to linearized scores. [file 12955_2024_2236_MOESM2_ESM.docx]

| APPENDIX. Translation of raw scores. score locations to linearized scores. | | | | | | | | |
| --- | --- | --- | --- | --- | --- | --- | --- | --- |
| Knowledge | | | |  | **Ability** | | | |
| Raw score | Score location | Standard Error | Linearized score |  | Raw score | Score location | Standard Error | Linearized score |
| 0 | -5.815 | 1.213 | 0 |  | 0 | -6.073 | 1.226 | 0 |
| 1 | -5 | 0.866 | 4 |  | 1 | -5.243 | 0.878 | 4 |
| 2 | -4.437 | 0.686 | 7 |  | 2 | -4.665 | 0.697 | 7 |
| 3 | -4.048 | 0.592 | 9 |  | 3 | -4.262 | 0.603 | 9 |
| 4 | -3.743 | 0.531 | 10 |  | 4 | -3.946 | 0.543 | 10 |
| 5 | -3.489 | 0.491 | 12 |  | 5 | -3.681 | 0.501 | 12 |
| 6 | -3.268 | 0.461 | 13 |  | 6 | -3.451 | 0.471 | 13 |
| 7 | -3.071 | 0.438 | 14 |  | 7 | -3.244 | 0.448 | 14 |
| 8 | -2.891 | 0.42 | 15 |  | 8 | -3.056 | 0.43 | 15 |
| 9 | -2.725 | 0.405 | 16 |  | 9 | -2.882 | 0.415 | 16 |
| 10 | -2.569 | 0.393 | 16 |  | 10 | -2.718 | 0.403 | 17 |
| 11 | -2.422 | 0.383 | 17 |  | 11 | -2.563 | 0.394 | 17 |
| 12 | -2.281 | 0.375 | 18 |  | 12 | -2.414 | 0.386 | 18 |
| 13 | -2.145 | 0.369 | 19 |  | 13 | -2.271 | 0.379 | 19 |
| 14 | -2.014 | 0.363 | 19 |  | 14 | -2.131 | 0.374 | 19 |
| 15 | -1.886 | 0.358 | 20 |  | 15 | -1.995 | 0.37 | 20 |
| 16 | -1.76 | 0.355 | 20 |  | 16 | -1.862 | 0.367 | 21 |
| 17 | -1.637 | 0.352 | 21 |  | 17 | -1.73 | 0.364 | 21 |
| 18 | -1.516 | 0.349 | 22 |  | 18 | -1.6 | 0.362 | 22 |
| 19 | -1.396 | 0.348 | 22 |  | 19 | -1.47 | 0.361 | 23 |
| 20 | -1.277 | 0.346 | 23 |  | 20 | -1.341 | 0.36 | 23 |
| 21 | -1.158 | 0.345 | 24 |  | 21 | -1.213 | 0.36 | 24 |
| 22 | -1.04 | 0.345 | 24 |  | 22 | -1.084 | 0.36 | 25 |
| 23 | -0.923 | 0.344 | 25 |  | 23 | -0.955 | 0.36 | 25 |
| 24 | -0.805 | 0.344 | 25 |  | 24 | -0.826 | 0.36 | 26 |
| 25 | -0.687 | 0.345 | 26 |  | 25 | -0.696 | 0.361 | 26 |
| 26 | -0.569 | 0.345 | 26 |  | 26 | -0.566 | 0.362 | 27 |
| 27 | -0.45 | 0.346 | 27 |  | 27 | -0.435 | 0.362 | 28 |
| 28 | -0.33 | 0.347 | 28 |  | 28 | -0.303 | 0.363 | 28 |
| 29 | -0.21 | 0.348 | 28 |  | 29 | -0.171 | 0.363 | 29 |
| 30 | -0.088 | 0.349 | 29 |  | 30 | -0.039 | 0.364 | 30 |
| 31 | 0.034 | 0.351 | 30 |  | 31 | 0.094 | 0.365 | 30 |
| 32 | 0.157 | 0.352 | 30 |  | 32 | 0.227 | 0.365 | 31 |
| 33 | 0.281 | 0.354 | 31 |  | 33 | 0.361 | 0.366 | 32 |
| 34 | 0.407 | 0.355 | 31 |  | 34 | 0.495 | 0.366 | 32 |
| 35 | 0.533 | 0.357 | 32 |  | 35 | 0.629 | 0.367 | 33 |
| 36 | 0.661 | 0.358 | 33 |  | 36 | 0.763 | 0.367 | 34 |
| 37 | 0.79 | 0.36 | 33 |  | 37 | 0.898 | 0.368 | 34 |
| 38 | 0.92 | 0.362 | 34 |  | 38 | 1.033 | 0.368 | 35 |
| 39 | 1.051 | 0.363 | 35 |  | 39 | 1.168 | 0.369 | 36 |
| 40 | 1.183 | 0.365 | 35 |  | 40 | 1.304 | 0.37 | 36 |
| 41 | 1.317 | 0.368 | 36 |  | 41 | 1.441 | 0.371 | 37 |
| 42 | 1.453 | 0.37 | 37 |  | 42 | 1.578 | 0.373 | 38 |
| 43 | 1.59 | 0.373 | 37 |  | 43 | 1.717 | 0.375 | 38 |
| 44 | 1.73 | 0.377 | 38 |  | 44 | 1.857 | 0.378 | 39 |
| 45 | 1.872 | 0.381 | 39 |  | 45 | 1.999 | 0.381 | 40 |
| 46 | 2.017 | 0.385 | 40 |  | 46 | 2.144 | 0.385 | 40 |
| 47 | 2.165 | 0.391 | 40 |  | 47 | 2.291 | 0.39 | 41 |
| 48 | 2.318 | 0.397 | 41 |  | 48 | 2.443 | 0.396 | 42 |
| 49 | 2.477 | 0.405 | 42 |  | 49 | 2.6 | 0.403 | 43 |
| 50 | 2.642 | 0.415 | 43 |  | 50 | 2.762 | 0.412 | 43 |
| 51 | 2.815 | 0.426 | 44 |  | 51 | 2.933 | 0.423 | 44 |
| 52 | 2.998 | 0.44 | 45 |  | 52 | 3.113 | 0.436 | 45 |
| 53 | 3.195 | 0.458 | 46 |  | 53 | 3.306 | 0.454 | 46 |
| 54 | 3.409 | 0.48 | 47 |  | 54 | 3.515 | 0.475 | 47 |
| 55 | 3.647 | 0.509 | 48 |  | 55 | 3.748 | 0.504 | 48 |
| 56 | 3.919 | 0.549 | 49 |  | 56 | 4.013 | 0.545 | 50 |
| 57 | 4.241 | 0.609 | 51 |  | 57 | 4.328 | 0.602 | 51 |
| 58 | 4.649 | 0.702 | 53 |  | 58 | 4.727 | 0.695 | 53 |
| 59 | 5.232 | 0.883 | 56 |  | 59 | 5.3 | 0.875 | 56 |
| 60 | 6.066 | 1.231 | 60 |  | 60 | 6.123 | 1.223 | 60 |

Online tool for logit transformation: Ekstrand J, Westergren A, Årestedt K, Hellström A, Hagell P. (2022) Transformation of Rasch model logits for enhanced interpretability. BMC Medical Research Methodology. Doi:  https://doi.org/10.1186/s12874-022-01816-1.
